# The Flavoproteins CryD and VvdA Cooperate with the White Collar Protein WcoA in the Control of Photocarotenogenesis in *Fusarium fujikuroi*

**DOI:** 10.1371/journal.pone.0119785

**Published:** 2015-03-16

**Authors:** Marta Castrillo, Javier Avalos

**Affiliations:** Department of Genetics, University of Seville, Sevilla, Spain; Karlsruhe Institute of Technology, GERMANY

## Abstract

Light stimulates carotenoid biosynthesis in the ascomycete fungus *Fusarium fujikuroi* through transcriptional activation of the structural genes of the pathway *carRA*, *carB*, and *cart*, but the molecular basis of this photoresponse is unknown. The *F*. *fujikuroi* genome contains genes for different predicted photoreceptors, including the WC protein WcoA, the DASH cryptochrome CryD and the Vivid-like flavoprotein VvdA. We formerly found that null mutants of *wcoA*, *cryD* or *vvdA* exhibit carotenoid photoinduction under continuous illumination. Here we show that the wild type exhibits a biphasic response in light induction kinetics experiments, with a rapid increase in carotenoid content in the first hours, a transient arrest and a subsequent slower increase. The mutants of the three photoreceptors show different kinetic responses: the *wcoA* mutants are defective in the rapid response, the *cryD* mutants are affected in the slower response, while the fast and slow responses were respectively enhanced and attenuated in the *vvdA* mutants. Transcriptional analyses of the *car* genes revealed a strong reduction of dark and light-induced transcript levels in the *wcoA* mutants, while minor or no reductions were found in the *cryD* mutants. Formerly, we found no change on *carRA* and *carB* photoinduction in *vvdA* mutants. Taken together, our data suggest a cooperative participation of WcoA and CryD in early and late stages of photoinduction of carotenoid biosynthesis in *F*. *fujikuroi*, and a possible modulation of WcoA activity by VvdA. An unexpected transcriptional induction by red light of *vvdA*, *cryD* and *carRA* genes suggest the participation of an additional red light-absorbing photoreceptor.

## Introduction

**Fig 1:**
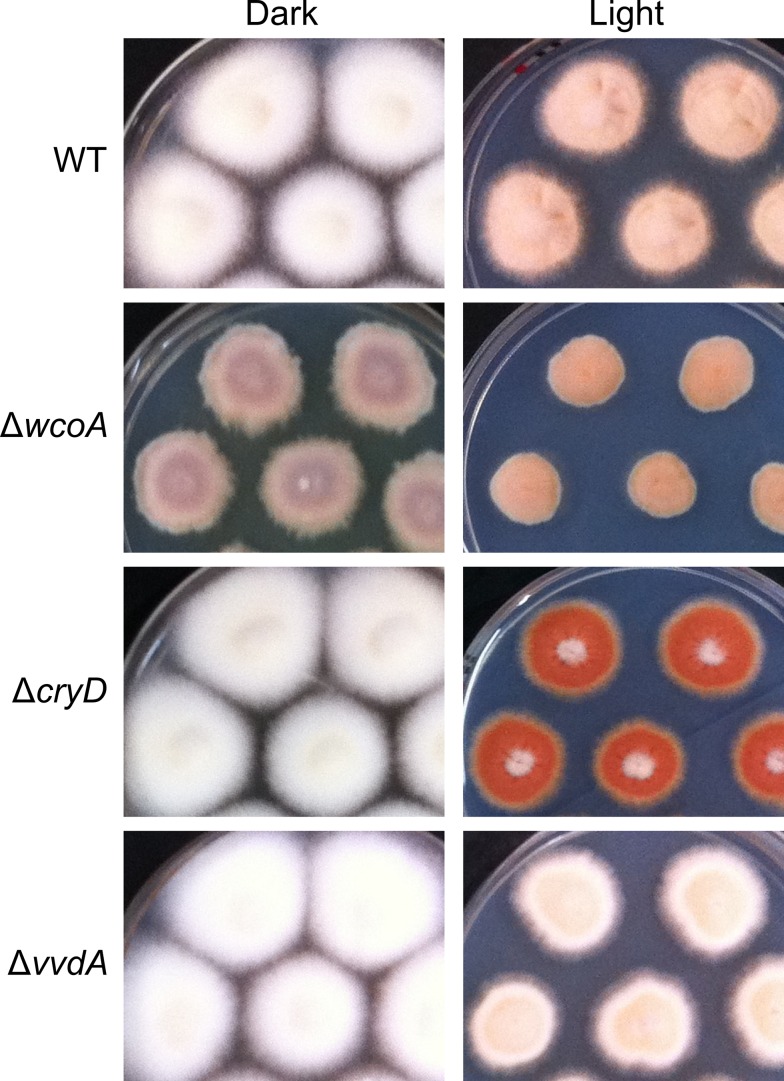
Colonies of the wild type and representative Δ*wcoA* (SF226), Δ*cryD* (SF236) and Δ*vvdA* (SF258) mutants. The strains were grown for 7 days at 30°C in the dark or under continuous illumination on DGasn medium. The brownish color of the Δ*wcoA* mutants in the dark and the reddish color of the Δ*cryD* mutants in the light are due to the production of secondary metabolite pigments unrelated to carotenoids, such as bikaverins and other polyketides.

Filamentous fungi are a widespread group of lower eukaryotes able to grow in a large diversity of natural habitats, where they use light as a key environmental signal to modulate physiological and developmental processes. Some fungi stand out for their ability to produce a wide range of secondary metabolites [1]. Species of the genus *Fusarium*, an ubiquitous group of phytopathogenic fungi able to produce an extensive array of compounds, belong to this class of fungi [2]. A representative example is *Fusarium fujikuroi*, well known for its capacity to produce gibberellins (GAs), growth-promoting plant hormones with biotechnological applications [3]. This fungus produces many other compounds, including pigments that provide characteristic colors to their mycelia [4]. When grown in the light, *F*. *fujikuroi* colonies acquire a characteristic orange pigmentation due to the synthesis of carotenoids (see wild type in Fig. 1), with the xanthophyll neurosporaxanthin as major component [5]. The biosynthetic carotenoid pathway of this fungus has been investigated in detail, and all the enzymatic genes have been identified and characterized [6–9].

The regulation of carotenoid biosynthesis has been object of special attention in several fungi, including *F*. *fujikuroi* [10]. Light was found to induce the synthesis of carotenoids in different *Fusarium* species [11]. This photoinduction was first investigated in *Fusarium aquaeductuum* [12,13], and later described in *F*. *fujikuroi* [14], *F*. *verticillioides* [15] and *F*. *oxysporum* [16]. A second regulatory signal is nitrogen availability, which plays a central role in the control of secondary metabolism in this fungus [17–19], including a negative effect on the production of carotenoids [20]. The only regulatory gene identified affecting carotenoid biosynthesis in *Fusarium* is *carS*, whose mutation leads to transcriptional up-regulation of the structural *car* genes [20,21], and accumulation of a high amount of carotenoids under different culture conditions [20,22]. *carS* encodes a protein of the ring finger family [16,23], whose molecular mechanism of action remains to be established.

Photoinduction of carotenogenesis has been investigated in detail in *Neurospora crassa*, where it is mediated by the heterodimeric White Collar (WC) complex [24]. The WC complex is activated by light through the WC-1 flavin-binding LOV/PAS domain and binds upstream promoter sequences of light-regulated genes to induce their transcription [25]. Similar WC complexes control diverse photoresponses in other fungi [26], including photoinduction of β-carotene production in the mucorales *P*. *blakesleeanus* and *M*. *circinelloides*. Against the predictions, the mutational loss of the WC-1-like protein WcoA of *F*. *fujikuroi* [27] or Wc1 of *F*. *oxysporum* [28] did not impede the photoinduction of the carotenoid pathway in these species, indicating the participation of other photoreceptors. A detailed action spectrum for photocarotenogenesis in *Fusarium aquaeductuum* was consistent with the participation of a flavin-based photoreceptor [12]. Besides the WcoA/Wc1 protein, the *Fusarium* genomes contain genes for three other presumptive flavin photoreceptors with counterparts in *N*. *crassa*, a small VIVID-like protein, a photolyase, and a DASH-cryptochrome [11]. Additionally, the *F*. *fujikuroi* genome includes a gene coding for a cryptochrome/photolyase, *cry1* (B. Tudzysnki, unpublished), with no ortholog in *N*. *crassa*.

Cryptochromes are blue light/UV-A photoreceptors possibly evolved from photolyases, able to bind two chromophores, flavin adenine dinucleotide (FAD) and pterin 5,10-methenyltetrahydrofolate (MTHF) [29,30]. Most cryptochromes have C-terminal extensions that are absent in photolyases, which play regulatory roles related to light control of growth, development, cell signaling or circadian rhythm in different taxonomic groups [30]. DASH-cryptochromes (abbreviated hereafter as cry-DASHs), a subgroup in this family, differ from cryptochromes in their ability to repair DNA lesions in single-stranded DNA [31] or loop-structures of duplex DNA [32]. A recent functional analysis of the *F*. *fujikuroi* cry-DASH gene *cryD* showed that it is strongly induced by light in the wild type but not in Δ*wcoA* disruption mutants [33]. Targeted Δ*cryD* mutants exhibited light-dependent alterations in the production of some secondary metabolites, as bikaverin and gibberellins, but they were not apparently affected in the synthesis of carotenoids.

Vivid-like photoreceptors are small flavin binding proteins represented by VVD (VIVID) of *N*. *crassa* and ENV1 (ENVOY) of *Hypocrea jecorina*. In *N*. *crassa*, VVD participates in photoadaptation of light-regulated genes [34,35], a function achieved through the modulation of the activity of the WC complex [36]. As a consequence, targeted Δ*vvd* mutants exhibit a deeper pigmentation and accumulate more carotenoids under light than the wild type [37,38]. ENV1 plays a more relevant role in *H*. *jecorina*, as indicates the drastic effect of the *env1* mutation on growth and morphology under light [39]. Additionally, ENV1 was found to participate in the regulation by light of cellulose degradation [39], sexual cycle [40], and photoadaptation of some light-induced genes [41]. Our recent investigation of the *vvd* gene of *F*. *fujikuroi*, *vvdA*, revealed significant differences in its function with VVD and ENV1: as found for *cryD*, *vvdA* expression is strongly induced by light in a WcoA-dependent manner, but the Δ*vvdA* mutants produced less carotenoids than the control strains and exhibited light-dependent alterations in conidiation and mycelia development [42]. However, expression analyses found no alterations in photoinduction or photoadaptation of the *car* genes.

Here we report on the role of the WcoA, CryD and VvdA photoreceptors in the regulation by light of carotenoid biosynthesis in *F*. *fujikuroi*. We found that this light response has two separate components in this fungus, a fast response in the first hours of illumination and a slower response upon more prolonged light exposure. The kinetics of the photoresponse in mutants for these putative photosensors indicates that WcoA is the major photoreceptor in *F*. *fujikuroi* carotenogenesis, and that CryD mediates a slower and less sensitive photoresponse, which explains the photoinduction exhibited by the *ΔwcoA* mutants under continuous illumination. The kinetics of the response is also altered by the loss of VvdA, which seems to play negative and positive effects on WcoA and CryD functions, respectively. Our results represent the first report on the participation of a cryptochrome in the regulation of carotenoid biosynthesis in any microorganism, and point to a complex organization of the light-detection machinery that controls carotenoid biosynthesis in *Fusarium*.

## Results

### Effect of *wcoA*, *cryD*, and *vvdA* mutations on regulation of carotenogenesis under constant illumination

**Fig 2:**
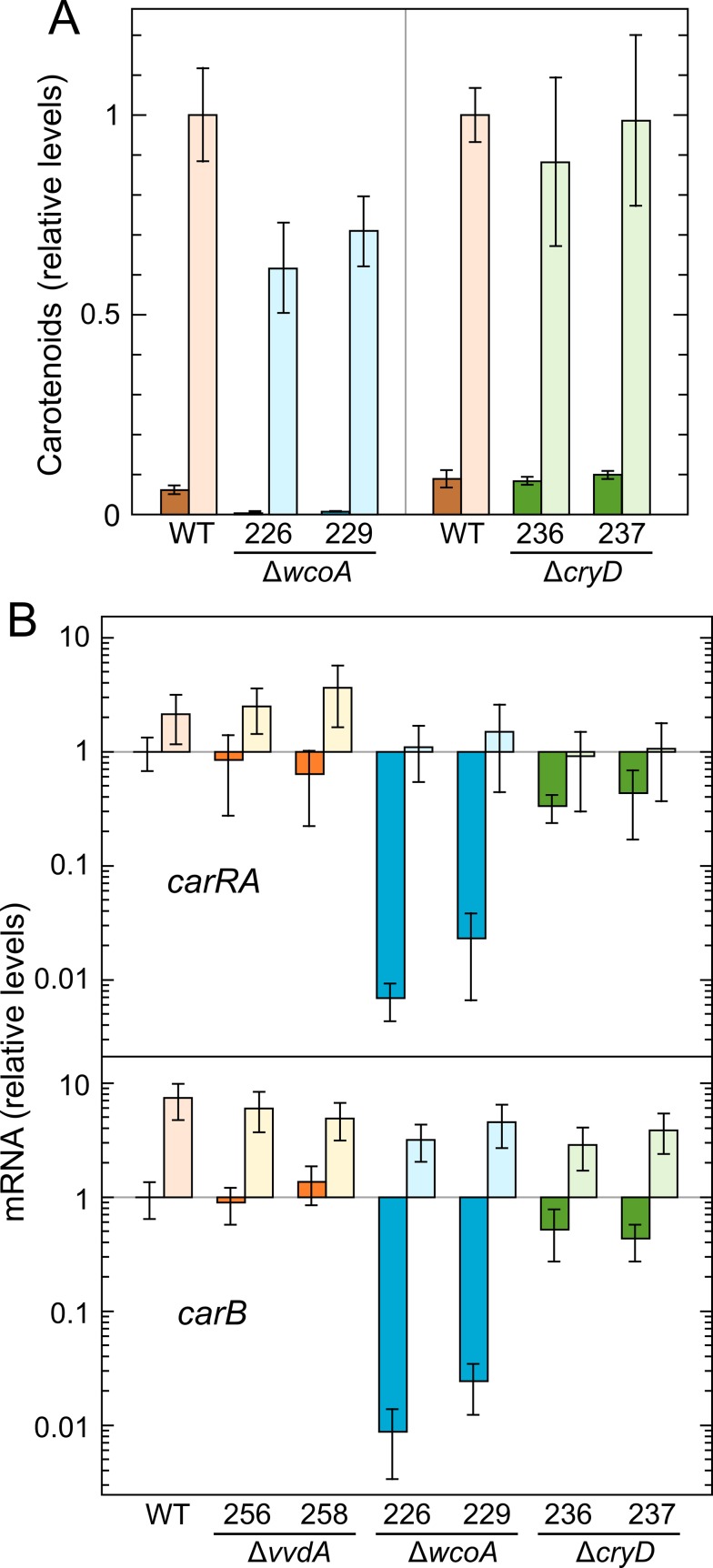
Effect of the mutation of the photoreceptor genes *cryD*, *wcoA* and *vvdA* on carotenogenesis in the dark versus constant illumination. A: Total concentration of carotenoids in the wild type and in parallel cultures of the Δ*wcoA* and Δ*cryD* mutants grown for 7 days at 30°C on DGasn medium in the dark or under continuous illumination. Data are the means and standard deviations of four determinations from two biological replicates, taking as 1 the value of the wild type under light. Data for Δ*vvdA* under the same culture conditions were recently published [42]. B: Real-time RT-PCR analyses of the genes *carRA* and *carB* in RNA samples of the wild-type and the indicated strains grown for 7 days at 30°C on DGasn medium in the dark or under continuous illumination. Relative expression for each gene was referred to the value in the wild type grown in the dark. Data are the means and standard deviations of six determinations from two biological replicates. Here, and in following figures, the wild type and the mutants for each gene are represented by different colors (brown, wild type; reddish-orange, Δ*vvdA;* blue, Δ*wcoA;* green, Δ*cryD*). Lighter versions of the colors are used for illuminated samples. Statistical analysis for differences between data of wild type and mutants are displayed in S1 Table.

The mutants of the genes for the predicted photoreceptors WcoA, CryD and VvdA exhibit differences in pigmentation and morphology (7-day old cultures shown in Fig. 1). Our former studies on these mutants included carotenoid analyses that showed the persistence of photoinduction in all the tested strains. Thus, Δ*vvdA* mutants accumulated in the light about 55–60% of the carotenoids produced by the wild type, while the synthesis in the dark was unaffected [42]. On the other hand, the Δ*wcoA* and Δ*cryD* mutants accumulated similar amounts of carotenoids in the light compared to the wild type [27,33]. However, in the case of the Δ*wcoA* mutants the experiment was done under different culture conditions and carotenoid levels in the dark were not determined. For more reliable comparison, we performed new carotenoid analyses of the Δ*wcoA* and Δ*cryD* mutants under the same conditions, 7-day incubations in the dark or under constant illumination (Fig. 2A). As recently found for the Δ*vvdA* strains [42], the Δ*wcoA* colonies produced less carotenoids than those of the wild type, albeit they maintained a clear photoinduction. Interestingly, the amounts of carotenoids in the dark in the Δ*wcoA* mutants were remarkably low compared to those of the wild type, suggesting a regulatory role for WcoA in the dark. However, in agreement with former observations [33], the total amount of carotenoids in the Δ*cryD* mutants remained very similar to those of the wild type, either in the dark or in the light.

To correlate these data with the expression of the genes of the carotenoid pathway, we determined the *carRA* and *carB* mRNA levels under the same culture conditions (Fig. 2B). The results showed a significant down-regulation of *carRA* and *carB* transcripts in the dark in the Δ*wcoA* mutants, consistent with their lower carotenoid content *in vivo*. A significant *carB* photoinduction, ranging from 3- to 7-fold compared to wild-type dark levels, was observed in all the strains tested. In the case of *carRA*, the increase was hardly detectable in the wild-type and Δ*vvdA* strains (2 to 3-fold), and inexistent in the Δ*wcoA* and Δ*cryD* mutants. However, because of their lower mRNA levels in the dark, *carRA* photoinduction was also manifest in these strains, most especially in the Δ*wcoA* mutants. In summary these experiments indicate the occurrence of light responses of *carRA* and *carB* expression in the mutants for the three photoreceptors, with differences in their dark and light levels.

### Effect of Δ*wcoA*, Δ*cryD*, and Δ*vvdA* mutations on kinetics of carotenoid accumulation upon illumination of dark-grown mycelia

**Fig 3:**
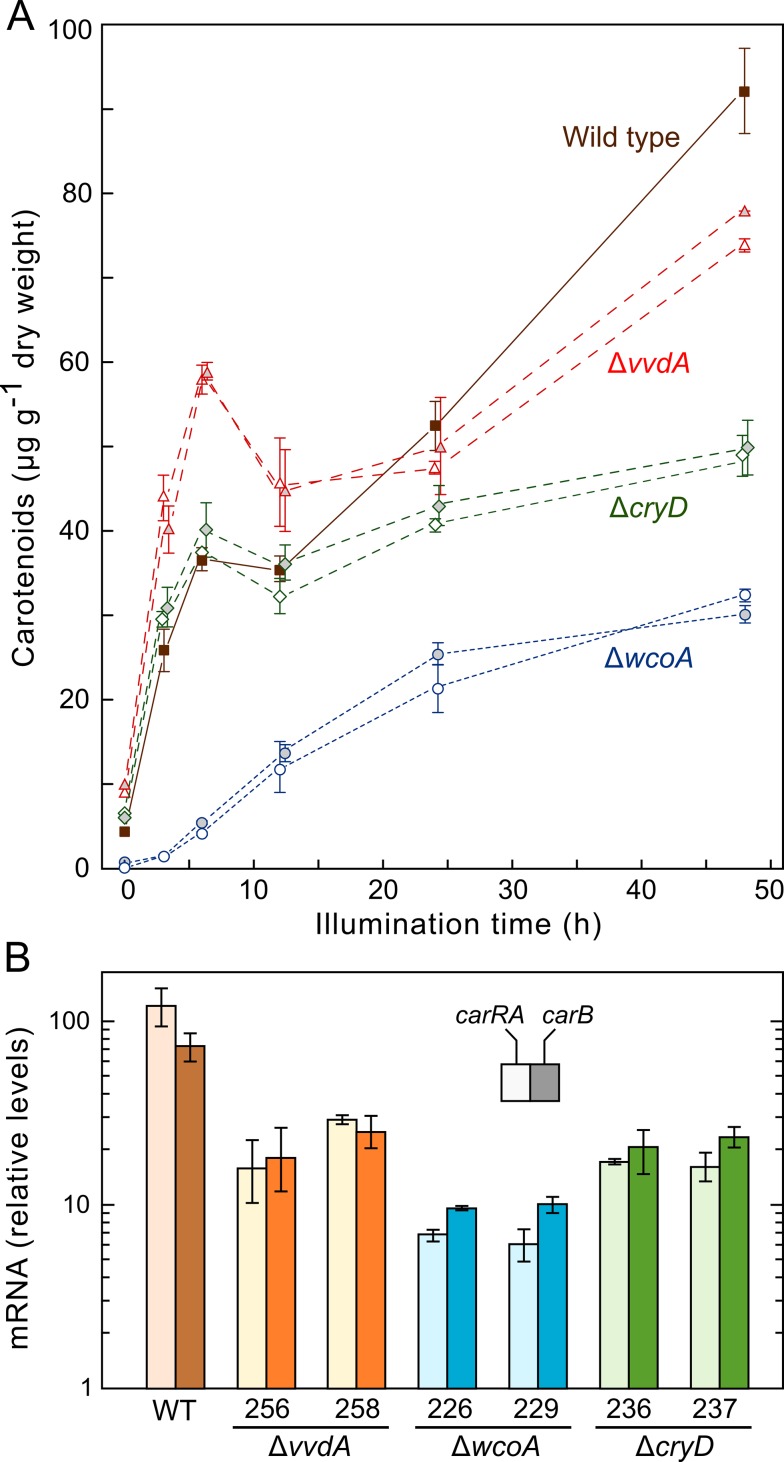
Light-induced carotenogenesis in wild type and mutants of the photoreceptor genes *cryD*, *wcoA* and *vvdA*. A: Kinetics of carotenoid accumulation after illumination of the wild type, Δ*wcoA* mutants SF226 and SF229, Δ*cryD* mutants SF236 and SF237, and Δ*vvdA* mutants SF256 and SF258. The strains were incubated for three days in the dark on DGasn agar and exposed to white light for the time indicated in abscissae. Each point is the mean and standard deviation of four determinations from two biological replicates. In this and in other figures, overlapping data were separated for better visualization. B: Real-time RT-PCR analyses of the genes *carRA* (pale bars) and *carB* (dark bars) in RNA samples of the same strains under the same culture conditions after 48 h illumination. Relative expression for each gene was referred to the value in the wild type grown in the dark. Data are the means and standard deviations of six determinations from two biological replicates.

The lower amounts of carotenoids in the light in the Δ*wcoA* and Δ*vvdA* mutants and the differences observed in *carRA* and *carB* mRNA levels in the Δ*wcoA* and Δ*cryD* mutants led us to investigate in more detail the responses of these strains to light. The wild type and the mutants for each presumed photoreceptor were incubated in parallel for three days in the dark, and illuminated afterwards up to 48 hours. Time-course of light-induced carotenoid accumulation was determined for each strain (Fig. 3A). Since the colonies grow further during illumination, the analysis was restricted to the central part of the colonies, i.e., the mycelium formed during the three days in the dark (see Materials and Methods). The results showed a biphasic response for the wild-type strain, with a rapid increase in the carotenoid content in the first 6 hours (first stage) followed by an arrest of the synthesis (6–12 h), and its resumption after a longer light exposure (12–48 h, second stage). The mutants for the photoreceptor genes showed different alterations of this pattern. Thus, the Δ*cryD* mutants responded to light as fast as the wild type in the first stage and paused the synthesis at the same time, but they exhibited a slower accumulation of carotenoids in the second stage. On the other hand, the response of the Δ*vvdA* mutants was faster in the first stage and slower in the second stage, but not as slow as in the Δ*cryD* mutants. In contrast with the former strains, the Δ*wcoA* mutants exhibited a much slower monophasic response, resulting after 48 h illumination in about 1/3 of the carotenoids accumulated by the wild type. In congruence with the data displayed in Fig. 2B the carotenoid content of the Δ*wcoA* mutants was much lower than those of the other strains at the start of illumination; so, paradoxically, the induction by light was proportionally higher in the Δ*wcoA* strains.

To gain more insights on the alterations of carotenoid photoinduction in the mutants, we determined the *carRA* and *carB* mRNA levels in the mycelia of the experiments described above after 48h illumination and the data were referred to the value of the wild-type strain in the dark (Fig. 3B). The mutants for the three photoreceptor genes exhibited a significant photoinduction of the transcript levels. However, the amounts were about 4-fold lower in the Δ*cryD* and Δ*vvdA* mutants and about 10-fold lower in the Δ*wcoA* mutants compared to those of the wild type. These data are consistent with the lower carotenoid accumulation exhibited by the different mutants in the later stage of the photoinduction kinetics.

Since *cryD* is transcriptionally photoinduced by WcoA [33], we checked the effect of expressing *cryD* in the Δ*wcoA* mutant in a WcoA-independent manner (S1 Fig.). For this purpose, the Δ*wcoA* mutant SF226 was transformed with a plasmid containing a tagged *cryD* ORF under control of the *gpdA* promoter of *A*. *nidulans*. After molecular analysis of several transformants, one strain was identified with the expected plasmid sequences (S1A Fig.) and exhibiting a strong *cryD* expression, with ca. 50-fold higher transcript levels in the dark and similar levels after one hour of illumination compared to the Δ*wcoA* control (S1B Fig.). The *cryD*-overexpressing strain exhibited a similar monophasic time-course of light-induced carotenoid accumulation as the control Δ*wcoA* mutant, but it accumulated about 70% more carotenoids (S1C Fig.), confirming the participation of CryD in photocarotenogenesis.

The results described above are consistent with a cooperative participation of the WcoA and CryD photoreceptors in the photoinduction of carotenogenesis in *F*. *fujikuroi*. To confirm this hypothesis, experiments were carried out to obtain a double Δ*wcoA* Δ*cryD* mutant. Two plasmids were constructed to obtain the targeted disruption of *wcoA* by homologous recombination in a Δ*cryD* background. In one of them the *wcoA* coding sequence was replaced by the nitrate reductase gene *niaD*, and in the other it was replaced by a geneticin resistance cassette. For *niaD* selection, a *niaD* ^*–*^mutant was obtained from the Δ*cryD* mutant SF237 by chlorate resistance. After three transformation attempts of the *niaD* ^-^ SF237 derivative, none of the transformants obtained either through protoplasts (39 *niaD* ^+^ colonies tested from two independent transformations) or through a biolistc approach (7 *niaD* ^+^ colonies tested from one transformation), exhibited the expected Δ*wcoA* phenotypic pattern, i.e., purple pigmentation in the dark and reduced mycelial hydrophobicity, and PCR tests confirmed the presence of an intact *wcoA* gene. Five additional protoplast-mediated transformation experiments achieved upon geneticin resistance selection, in this case using SF236, led to the isolation of 16 transformants. All of them resulted from ectopic plasmid integrations, and no *wcoA* mutants were obtained. As an alternative approach, mutagenesis experiments were done with SF236 and SF237 and a total of 15 mutants with a Δ*wcoA*-like pigmentation were analyzed. The *wcoA* alleles from four mutants, those exhibiting a phenotype more similar to that of the *wcoA* mutant SF226, were cloned and sequenced, but no mutations were found in their respective *wcoA* coding sequences. Taken together, these results suggest a lack of viability for the double Δ*wcoA* Δ*cryD* mutant.

### Effect of light intensity on photoinduction of carotenogenesis in Δ*wcoA*, Δ*cryD*, and Δ*vvdA* mutants

**Fig 4:**
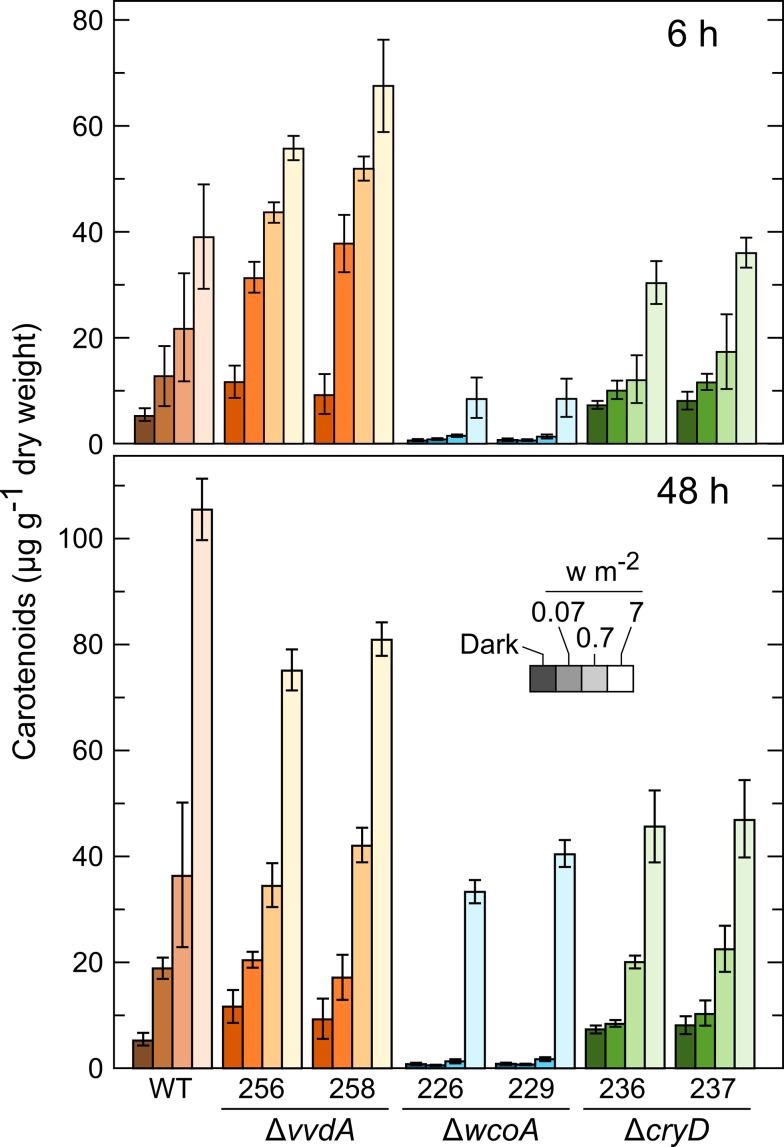
Effect of light intensity on photoinduction of carotenoid accumulation in the wild type and Δ*wcoA* mutants SF226 and SF229, Δ*cryD* mutants SF236 and SF237, and Δ*vvdA* mutants SF256 and SF258. The strains were incubated for three days in the dark in DGasn medium (left bars, darker colors) and exposed to 0.07 W m^-2^, 0.7 W m^-2^ or 7 W m^-2^ (brighter colors from left to right) of white light for 6 hours (above) and 48 h (below). Data show means and standard deviations of four determinations from two biological replicates. Statistical analysis for differences between data of wild type and mutants are displayed in S1 Table.

The predicted photoreceptors under investigation may differ in their sensitivities to light. To obtain more information on the alterations resulting from the loss of each photoreceptor we used neutral filters to reduce the light intensity in the photoinduction kinetics experiments. To distinguish the effects on the fast and slow stages of the photoresponse (Fig. 3A), we determined the carotenoid content in the mycelia in the dark and after 6 h or 48 h illumination under 0.07, 0.7 and 7 W m^-2^ (Fig. 4 and S2 Fig.). The results confirmed the different responses of the mutants after the first and second kinetics stages. After 6 h illumination, the Δ*cryD* mutants contained similar amounts of carotenoids than those found in the wild type while the Δ*wcoA* mutants produced lower amounts. On the other hand, after 48 h the Δ*cryD* mutants produced less carotenoids than the wild type, but their levels were similar to those of the Δ*wcoA* mutants. Also, the carotenoid content of the Δ*vvdA* strains was higher than those of the wild type after 6 h, and lower after 48h, indicating that these mutants accumulate less carotenoids than the wild type upon prolonged incubation, as found in the cultures under constant illumination [42].

The results were consistent with differences in light sensitivity between different photoreceptors. No significant photoinduction was found in the Δ*wcoA* mutants under 0.07 and 0.7 W m^-2^, indicating that CryD is able to respond only to high light intensities. However, the Δ*cryD* mutants showed a significant photoinduction even at the lower light intensity, consistent with a high sensitivity to light of the WcoA photoreceptor. Interestingly, after 6 h illumination the response to low or very low light intensity was enhanced in the Δ*vvdA* mutants compared to the wild type. Thus, these mutants accumulated more carotenoids after this time under 0.7 W m^-2^ than the wild type under 7 W m^-2^.

**Fig 5:**
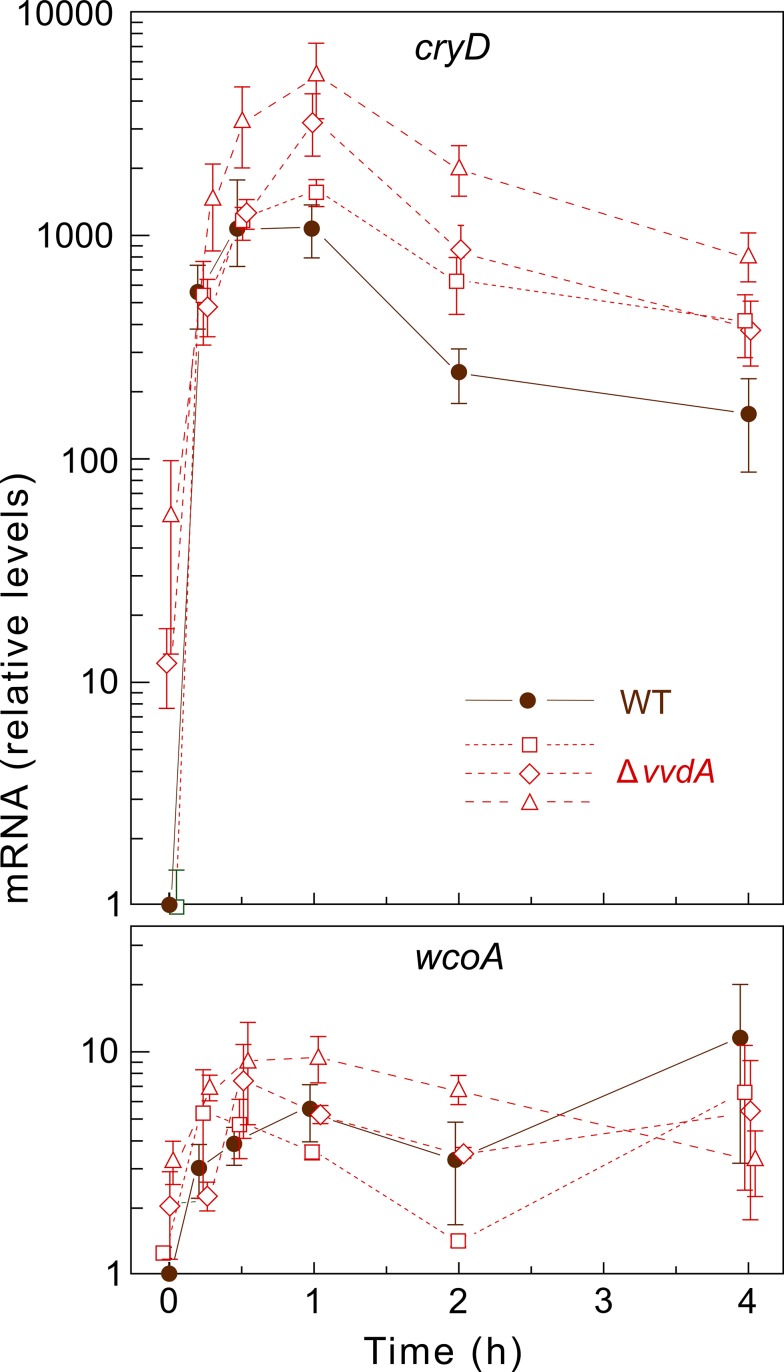
Effect of the *vvdA* mutation on expression of the photoreceptor genes *cryD* and *wcoA*. Real-time RT-PCR analyses of genes *cryD* and *wcoA* in total RNA samples from the wild type (brown squares) and the Δ*vvdA* mutants SF256 (red circles), SF257 (red rhombs) and SF258 (red triangles) grown for three days in DGasn medium in the dark or after 15 min, 30 min, 1 h, 2 h, or 4 h exposure to 7 W m^-2^ white light. Relative levels are referred to the value of the wild type in the dark. Data show means and standard deviations for nine measurements from three biological replicates. Statistical analysis for differences between data of wild type and mutants are displayed in S1 Table.

To test if the function of VvdA is performed on transcription of the other photoreceptor genes, we determined the effect of the Δ*vvdA* mutation on *cryD* and *wcoA* mRNA levels. For more complete information, in this case the analysis was done with three independent Δ*vvdA* mutants (Fig. 5). The results confirmed the strong photoinduction of *cryD* mRNA, as formerly reported [33]. However, the *cryD* mRNA levels were visibly enhanced in the Δ*vvdA* strains compared to the wild type. As already found for *carRA* and *carB* [33], the Δ*vvdA* mutants exhibited a similar photoadaptation of *cryD* mRNA levels compared to wild type. On the other hand, a minor photoinduction was found for *wcoA* in the same samples. This induction, which was disregarded in former analysis [27], was basically unaffected in the *vvdA* mutants.

### Effect of the mutation of the *wcoA* and *cryD* genes on expression of photoregulated genes

**Fig 6:**
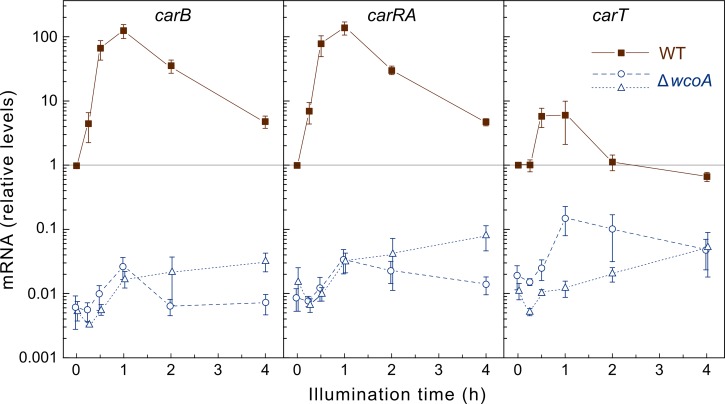
Effect of the *wcoA* mutation on expression of genes of the carotenoid pathway. Real-time RT-PCR analyses of genes *carB*, *carRA* and *carT* in total RNA samples from the wild type (brown squares) and the Δ*wcoA* mutants SF226 (blue circles) and SF229 (blue triangles) grown for three days in DGasn medium in the dark or after 15 min, 30 min, 1 h, 2 h, or 4 h exposure to 7 W m^-2^ white light. Relative levels are referred to the value of the wild type in the dark. Data show means and standard deviations for nine measurements from three biological replicates.

Recently, it was shown that the mutation of the *vvdA* gene does not affect the photoinduction pattern of the structural genes *carRA* and *carB* [42]. For better understanding of the phenotypic effects of the Δ*wcoA* and Δ*cryD* mutations on the photoinduction kinetics of carotenoid accumulation, we investigated the effect of light on *carRA*, *carB* and *carT* mRNA levels in these mutants in comparison to the wild type. Formerly, it was reported that the *carB* gene was down-regulated in *wcoA* mutants, while the result was less clear for *carRA* [27]. We have analyzed in more detail two Δ*wcoA* mutants, extending the analysis up to 4 h of illumination (Fig. 6). As expected, the RT-PCR data for the wild type showed a strong photoinduction of *carRA* and *carB* transcript levels, exceeding one hundred fold the levels in the dark after one hour, and decaying in the following hours. A more modest induction was exhibited by *carT*, which did not reach a tenfold increase, returning afterwards to background levels. In agreement with the lower carotenoid content of the Δ*wcoA* mutants in the dark (Figs. 2 and 3), the *car* mRNA levels were much lower in the dark in these strains than in the wild type. The photoinduction was also very low, with *carRA*, *carB* and *carT* transcript levels still much lower in the *wcoA* mutants after illumination than in the wild type in the dark. These results fit the slow accumulation of carotenoids by the Δ*wcoA* mutants in the kinetics experiments (Fig. 3A), which could be explained by a slow up-regulating activity of CryD.

**Fig 7:**
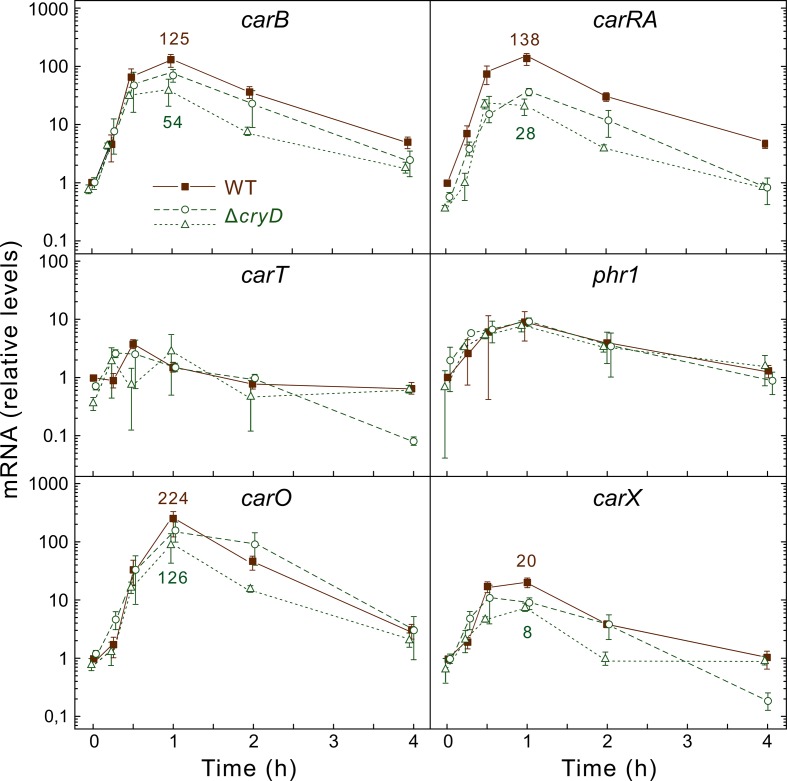
Effect of the *cryD* mutation on expression of genes of the carotenoid pathway and other light-regulated genes of *F*. *fujikuroi*. Real-time RT-PCR analyses of genes *carB*, *carRA*, *carT*, *carO*, *carX* and *phr1* in total RNA samples from the wild type (brown squares) and the Δ*cryD* mutants SF236 (green circles) and SF237 (green triangles) grown for three days in DGasn medium in the dark or after 15 min, 30 min, 1 h, 2 h, or 4 h exposure to 7 W m^-2^ white light. Relative levels are referred to the value of the wild type in the dark. Data show means and standard deviations for nine measurements from three biological replicates. Statistical analysis for differences between data of wild type and mutants are displayed in S1 Table. For better comparison, mean values for mRNA levels of genes *carB*, *carRA*, *carO* and *carX* in wild type and mutants at the point of maximal expression (one hour of illumination) are indicated in the figure.

In contrast to the Δ*wcoA* mutants, the expression of the genes *carRA* and *carB* was strongly photoinduced in the Δ*cryD* mutants. However, there was a decrease of at least five-fold in the photoinduction levels of *carRA* (Fig. 7), but this effect was less manifest on *carB* and hardly detectable for *carT*, that only reached a 4-fold induction in the wild type in this set of experiments. As found for *carB*, the Δ*cryD* mutation produced minor or no effects in the photoinduction of other light-regulated genes, such as *carO*, *carX* and *phr1*, encoding a rhodopsin protein [21], a retinal-producing carotenoid oxygenase [8], and a CPD-photolyase [43], respectively. On the other hand, no photoinduction was found for *carRA* and *carB* in the Δ*wcoA* mutant overexpressing *cryD* in the dark (S1C Fig.). Taken together, the results indicate that CryD does not play an important role in the transcriptional regulation of light-induced genes in *F*. *fujikuroi*.

### Effect of blue and red light on expression of *carRA* and photoreceptor genes

**Fig 8:**
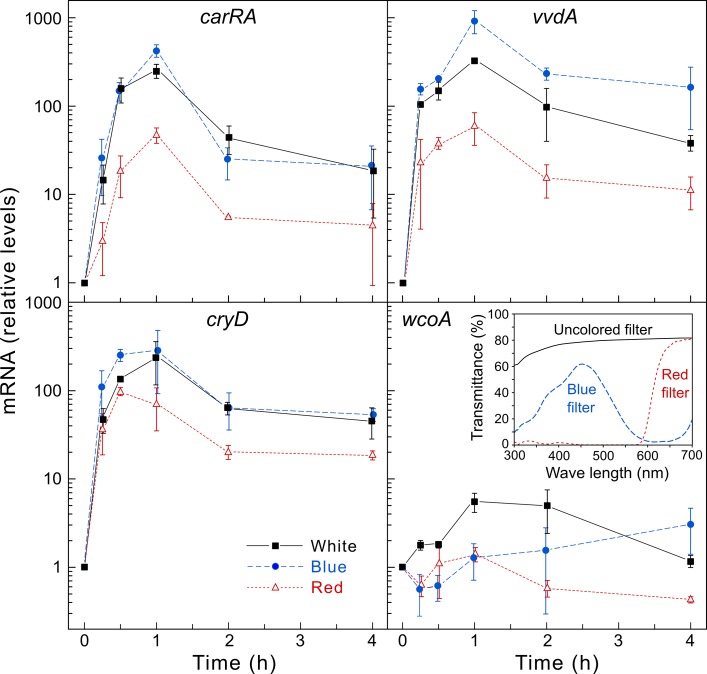
Effect of light wavelength on photoinduced expression of the photoreceptor genes *cryD*, *wcoA* and *vvdA*, and the carotenogenic gene *carRA*. Real-time RT-PCR analyses of genes *cryD*, *wcoA*, *vvdA* and *carRA* in total RNA samples from the wild type grown for three days in DGasn medium in the dark or after 15 min, 30 min, 1 h, 2 h, or 4 h exposure to white light (black symbols), blue light (blue symbols) or red light (red symbols). Relative levels are referred to the value of the wild type in the dark. Data show means and standard deviations for six measurements from two biological replicates. The inset shows the transmittance spectra of the uncolored, blue and red filters used for illumination in this experiment (adapted from [33]).

The Δ*cryD* mutants exhibit light-dependent alterations in morphology and pigmentation. However, these alterations were not observed under red light [33]. CryD, WcoA and VvdA are predicted blue-light photoreceptors. To check the dependence of *F*. *fujikuroi* photocarotenogenesis on light wavelength, we compared the effect of blue, red and white lights on transcript levels of the structural gene *carRA*, and on those of the photoreceptor genes *cryD*, *wcoA* and *vvdA* (Fig. 8). As expected blue light was as efficient as white light to induce the expression of the gene *carRA*; however, red light produced a significant photoinduction, with transcript levels reaching about 10% of the induction produced by white or blue light. A similar pattern was observed for the gene *vvdA*, with blue light giving the higher efficiency. Surprisingly, red light was particularly efficient to induce *cryD* mRNA levels, being as efficient as white light during the first 30 min of induction. In contrast, the minor induction of *wcoA* was less apparent either with blue or red light. Congruently with these results, the mycelia incubated for one week under red light contained about 54 μg g^-1^ dry weight compared to 7 μg g^-1^ dry weight in the dark or 197 μg g^-1^ dry weight under white light. Taken together, the data suggest the participation of a red-light photoreceptor in the transcriptional regulation of the genes for carotenogenesis and in those for the VvdA and CryD photoreceptors.

## Discussion

Induction of carotenogenesis by light is a well-known photoresponse in some filamentous fungi. Former data indicated that the regulatory mechanism for this photoinduction in *F*. *fujikuroi* differs from that of other fungal models, such as *N*. *crassa*, *P*. *blakesleeanus* and *M*. *circinelloides*, where photocarotenogenesis is mediated by a White Collar complex (reviewed by [44]). The persistence of photoinduction of carotenogenesis in the Δ*wcoA* mutants of *F*. *fujikuroi* [27] led us to investigate other predicted blue-light photoreceptors, as the cry-DASH CryD and the small flavoprotein VvdA. The loss of WcoA has a drastic effect on the ability of the *car* genes to respond to light, a result that contrast with the persistence of photocarotenogenesis in these mutants under constant illumination. Moreover, the down-regulatory effect of the Δ*wcoA* mutation is also noticeable in the dark, fitting former observations on light-independent functions for this protein [27]. The impact of the Δ*wcoA* mutation on *carRA* and *carB* expression was more drastic than in our former analyses [27]. This difference may be attributed to the higher resolution of the methodology used in this work, that led also to detect higher photoinduction levels in the wild type.

The drastic transcriptional effect of the *wcoA* mutation clearly differs from the milder effect of the *cryD* mutation, that produced an appreciable reduction in the mRNA levels of the gene *carRA*, but it did not prevent its photoinduction. This gene plays a key role in carotenoid biosynthesis, since it encodes the enzyme required for the synthesis of phytoene, the uncolored precursor of all carotenoids. However, the mutants lacking a functional CryD basically conserved the induction pattern of other light-regulated genes, including *carB*, and accumulated similar amounts of carotenoids than the wild type upon continuous illumination. The persistence of light-induced carotenogenesis in the Δ*cryD* and Δ*wcoA* mutants indicates that neither WcoA nor CryD act as single photoreceptors for this response.

For a better understanding of the photoinduction mechanism, we investigated the kinetics of carotenoid accumulation resulting from the illumination of young dark-grown colonies from the wild type and mutants of three different photoreceptors. The Δ*wcoA* and Δ*cryD* mutants exhibited alterations in the production of bikaverin [27,33], a red pigment that could interfere with the absorption of blue light by the putative photoreceptors, but the young colonies used in our experiments contain low concentrations of these pigments. These experimental conditions led us to identify two different induction stages: a rapid response achieved during the first 6 hours, and a subsequent slower response holding at least for 36 h. Former experiments with submerged cultures of *F*. *fujikuroi* showed simple induction kinetics, with a significant increase of the carotenoid content after 3 h illumination and the reach of maximum levels after 24 h [14]. Similar induction curves were described in submerged conditions for *F*. *aquaeductuum* [12] and *N*. *crassa* [45], although a significant photoinduction was detected in the latter after one-hour light exposure. A more detailed analysis revealed however a two-step kinetics response in *N*. *crassa*. Under similar light intensities to those used in our experiment (8 W m^-2^), a first induction step occurred in this fungus during the first minute of illumination [46], a result consistent with a rapid activity stimulation of an enzymatic set already available in the dark. A simple photoinduction curve of carotene accumulation was also observed in *P*. *blakesleeanus*, but fluence-response curves achieved with different wavelengths revealed a two-step response, suggesting the participation of two photosystems of different sensitivity thresholds [47]. The single mutants of the WC complex MadA/MadB still exhibit photoinduction, but the double mutants become blind [48], indicating that this WC complex is responsible for photocarotenogenesis in this fungus.

The alteration in the kinetics curves of carotenoid accumulation in mutants devoid of functional WcoA, CryD or VvdA proteins provide valuable clues on the participation of these predicted photoreceptors in carotenoid photoinduction in *F*. *fujikuroi*. The results are consistent with the participation of WcoA in a highly sensitive photoinduction mechanism, fast but transitory, while CryD is involved in a less sensitive but more sustained light-mediated stimulation. The first mechanism could be involved in the rapid response to sudden changes of light conditions and its subsequent adaption, as indicates the transience of the transcriptional photoinduction of the structural *car* genes. On the other hand, the latter mechanism could be involved in the response to persistent day-light illumination conditions. Both mechanisms seem to operate simultaneously under long standing illumination, as indicates the low but detectable increase in *carB* mRNA levels of the *car* genes in 7-day-old colonies of either the wild type or the Δ*wcoA*, Δ*cryD* or Δ*vvdA* mutants incubated under constant light. The low mRNA levels under these conditions suggest the operation of an efficient long-standing adaptation mechanism, in which VvdA apparently does not participate.

The phenotypic effect of the *cryD* mutation on carotenoid accumulation in *F*. *fujikuroi* is the first report on the participation of a cryptochrome on light regulation of carotenogenesis in any microorganism. The conservation of high photoinduction levels for the *car* genes in the Δ*cryD* mutants suggests that this regulation is achieved mostly through a non-transcriptional mechanism. The ability of cry-DASHs to bind nucleic acids [30], and the presence in the CryD C-terminal extension (CryD sequence: GenBank HE650104) of several RGG motifs (M. Castrillo et al., manuscript in preparation), formerly associated to RNA binding [49], is suggestive of a possible role for CryD promoting the stability or the translation of mRNAs for the *car* genes. Thus, a lower stability could explain the minor reductions observed in photoinduced *carRA* or *carB* mRNA levels in the Δ*cryD* mutants in Fig. 7. A similar mechanism could explain the lack of correlation between bikaverin biosynthesis and mRNA levels for the structural genes of the bikaverin pathway in the Δ*cryD* mutants grown in the light [33]. In contrast, the regulation of carotenogenesis by light in *N*. *crassa* appears to be under exclusive control of the WC complex, and no effect on this photoresponse was reported for the mutant of the *cryD* ortholog *cry-1* [50]. Another cry-DASH protein, Cry1, was investigated in *Sclerotinia sclerotium*. In this fungus, *cry1* transcripts are found at higher levels in light-exposed stages of apothecia development [51], but the *cry1* mutation has only a minor effect in this developmental process. Interestingly, *S*. *sclerotium* produces β-carotene and its production is induced by light coupled to sclerotia formation [52]. However, the consequences of the *cry1* mutation on light-induced β-carotene production in this fungus have not been reported.

The results support the participation of VvdA in the regulation of photocarotenogenesis in *F*. *fujikuroi*. As already mentioned, the *vvdA* mutants accumulate less carotenoids than the wild type under constant illumination [42]. As the wild type, the Δ*vvdA* mutants exhibit a biphasic photoresponse; however, the response is enhanced in its first stage and it is attenuated in the second stage. Moreover, the Δ*vvdA* mutants become more sensitive than the wild type under low light intensity. This result would be consistent with the function of VvdA as a negative modulator of WcoA activity, a regulatory scenario that reminds the down-regulation of WC-1 by VVD in *N*. *crassa* [36]. This is apparently contradicted by the lack of effect of the Δ*vvdA* mutation on the photoinduction of *carRA* and *carB* levels [42]. However, the latter experiments were achieved with submerged cultures while the carotenoid kinetics experiments were achieved on surface colonies. Differences in VvdA levels in the dark between both culture conditions might explain this discrepancy.

The slower response of the Δ*vvdA* mutants in the second stage of the induction kinetics could be explained by the interaction of VvdA with the WC complex or by a positive regulatory effect on CryD activity. In support of the first hypothesis, upon illumination VVD inactivates the WC complex in *N*. *crassa* forming a stable VVD-WCC heterodimer, which at the same time protects the light-induced WC complex from degradation [42]. It is very likely that a similar interaction occurs in *F*. *fujikuroi*, allowing the persistence of WC proteins that could eventually get free from VvdA to allow a slower photocarotenogenesis. Therefore, the light-activated WC complex would not be restrained by VvdA in the Δ*vvdA* mutant, leading to an enhanced carotenoid photoinduction in an early stage, but it could be more rapidly degraded afterwards, explaining the lower synthesis in a later stage. Taken together, the phenotypic effects of the *vvdA* mutation suggest that the participation of VvdA in carotenoid photoinduction is mainly achieved interfering with WcoA function, albeit a possible role on CryD function is not discarded.

**Fig 9:**
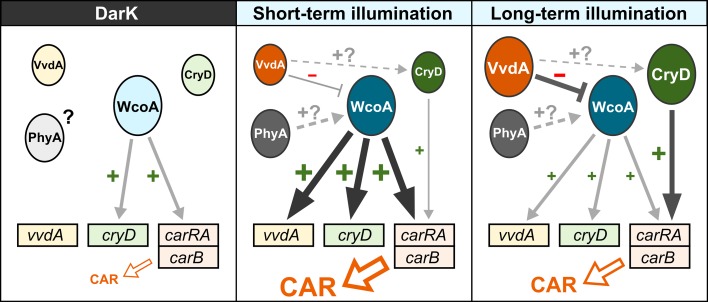
A hypothetic model for regulation of carotenogenesis by light in *F*. *fujikuroi*. Circles correspond to proteins and squares to genes. The sizes of the circles represent low or high protein amounts in the corresponding conditions. In the absence of information on PhyA regulation, the same size is used for this protein. Non-activated proteins are indicated in pale colors with black letters and light-activated proteins are indicated in dark colors with white letters. The thickness of the arrows roughly represents predicted intensity of the regulatory effects, while positive and negative signs (arrows and truncated lines) represent inducing or repressing effects. Effect of VvdA could be achieved by protein/protein interactions, while CryD would up-regulate the *car* genes post-transcriptionally. The final effect on carotenoid biosynthesis is indicated below.

In conclusion, the data provided in this work suggest a global model for carotenoid photoinduction in *F*. *fujikuroi* (Fig. 9). In this model, WcoA modulates the synthesis of carotenoids in the dark allowing a basal transcription rate for the structural genes, represented here by *carRA and carB*. A sudden light stimulus produces a rapid response in which WcoA is activated and promotes the expression of the *car* genes, together with those of *cryD* and *vvdA*. The activation of the *car* genes results in a rapid carotenoid accumulation. These WcoA functions are probably achieved as a WC complex with the WC-2-like partner WcoB, not represented in the scheme. However, this scenario changes with the persistence of the light stimulus in the following hours, during which light-activated VvdA restrains WcoA activity and light-activated CryD up-regulates *carB* and *carRA*, seemingly through a non-transcriptional mechanism rather than at transcription level. The coordinated participation of both photoreceptors explains the slow induction of carotenoids after illumination in the absence of WcoA and the slower response after prolonged illumination in the absence of CryD.

Upon sustained light exposure, VvdA keeps WcoA partially inactivated. However, WcoA would still mediate a certain transcriptional photoinduction in later stages, since carotenoid production is not totally prevented in Δ*cryD* mutants during long-term illumination. VvdA could play a role under these conditions preventing the inactivated WC complexes from degradation, thus contributing to a more persistent but attenuated photoinduction. Alternatively, VvdA could have a positive influence on CryD activity by an unknown molecular mechanism. Finally, the data suggest the participation of the only phytochrome found in the *Fusarium* proteomes [11], whose *F*. *fujikuroi* ortholog is called here PhyA (GenBank CCT69959). The activation of *carRA* transcripts by red light requires a functional WcoA, as indicates the drastic effect of the Δ*wcoA* mutation under white light, which includes red wavelengths. Tentatively, PhyA could act as an accessory red-light absorbing pigment that would transfer the signal to the putative WcoA/WcoB complex.

The regulatory system in *F*. *fujikuroi* reminds that of *N*. *crassa* in that a WC protein, susceptible to be activated by light and modulated by a VVD protein, is the only specific transcriptional activator that controls the expression of the structural genes of the carotenoid pathway. However, *F*. *fujikuroi* exhibits a more sophisticated regulation with some distinctive features: WcoA mediates a basal transcription level in the dark, allowing a detectable carotenoid biosynthesis under these conditions, and other photoreceptors add to WcoA to stimulate the synthesis in response to light, as CryD and probably also PhyA. This phytochrome could account for a supplementary response to red light, but its participation is just speculative and remains to be experimentally demonstrated in future works.

## Materials and Methods

### Strains and culture conditions

*Fusarium fujikuroi* FKMC1995 (Kansas State University Collection, Manhattan, KS, USA) was used as the wild-type strain. *F*. *fujikuroi* mutants for the *wcoA*, *cryD* and *vvdA* genes were previously described: *wcoA* mutants (SF226 and SF229), referred as Δ*wcoA*, were generated by interruption of the *wcoA* coding region with a hygR resistance cassette [27]. Δ*cryD* mutants (SF236 and SF237) and Δ*vvdA* mutants (SF256, SF257 and SF258) were obtained by replacement of the 5’ coding sequence [33] or the whole coding sequence [42], respectively, with the same hygR cassette. A strain constitutively expressing *cryD* was generated in this work and described in a later section.

DG minimal medium, that contains 3 g l^-1^ of NaNO_3_ as nitrogen source, has been previously described [53]. Unless otherwise stated, the strains were grown in DGasn medium, with the same composition of DG minimal medium except that the 3 g l^-1^ of NaNO_3_ were replaced by 3 g l^-1^ asparagine. Agar cultures were inoculated with sterile toothpicks in seven symmetrical positions on the agar surface, and liquid cultures were inoculated with 10^6^ fresh conidia. To obtain conidia, the strains were incubated on solid medium for 7 days at 22°C under indirect illumination. Conidia were collected by washing the agar surface with water; afterwards they were separated from mycelial debris by filtration, washed with water by centrifugation and counted in a hemocytometer (Bürker chamber, Blau Brand, Germany).

Illumination experiments were achieved under four fluorescent tubes Philips TL-D 18W/840, placed at a distance of ca. 60 cm. For carotenoid analysis the strains were grown at 30°C in darkness or under 7 W m^-2^ white light for the times indicated. For carotenoid kinetics assays the strains were grown on solid medium for 3 days in complete darkness at 30°C and exposed afterwards for 3, 6, 12, 24 or 48 h to 7 W m^-2^ white light, or for 6 or 48 h to 7 W m^-2^, 0.7 W m^-2^ or 0.07 W m^-2^ white light. Reduction of light intensities was achieved with neutral grey plastic filters, whose transmittance was determined spectrophotometrically. Since the colonies keep expanding after light onset, mycelial samples were restricted to the dark-grown colony areas, avoiding colony borders grown under continuous illumination. For this purpose, the border of the colonies after the 3-day dark incubation was marked on the back of the Petri dishes and mycelia out of the delimited area, grown during light exposure, was removed with a blade.

For light induction analyses of gene expression, the strains were grown in 15 Ø cm Petri dishes containing 80 ml of liquid medium. The dishes were incubated for 3 days at 30°C in darkness and illuminated afterwards with 7 W m^-2^ white light for 15, 30, 60, 120 and 240 minutes. When indicated, the light was filtered through transparent, blue or red cellophane sheets in appropriately sealed boxes, resulting in 6 W m^-2^, 1.5 W m^-2^, and 1.7 W m^-2^ respectively [33]. Expression analyses were also performed with mycelia from 7-day-old colonies grown either in the dark or under continuous illumination, or from colonies grown in the dark and illuminated with 7 W m^-2^ white light for 48 h.

### RT-PCR expression analyses

For expression assays in liquid media, the mycelial layer growing on the bottom of the plate was removed with a rod, immediately frozen in liquid nitrogen and stored at -80°C. For 7-day incubation experiments on solid medium, samples were collected from the surface of the colony with a sterile blade and immediately frozen in liquid nitrogen and stored at -80°C.

RNA was extracted with the RNeasy Plant Mini Kit (Qiagen, Chatsworth, CA, USA) and concentration was estimated with a Nanodrop ND-1000 spectrophotometer (Nanodrop Technologies, Wilmington, DE, USA). To avoid possible DNA contaminations, 2.5 μg RNA samples were treated with 10 u of DNase I (Affymetrix USB products, Santa Clara, CA, USA) for 15 min at 25°C, followed by 10 minutes of inactivation at 65°C. DNAse I-treated RNA samples were converted to cDNA with the Transcriptor First Strand cDNA synthesis Kit (Roche, Mannheim, Germany) and final concentrations were set to 25 ng μl^-1^. Quantitative expression analyses were performed on cDNA samples in a Lightcycler 480 equipment (Roche). For amplification and detection, LightCycler 480 SYBR Green I Master (Roche) was used following manufacturer reaction protocol. The genes analyzed in these studies and the corresponding forward and reverse primers used in the RT-PCR amplifications were *carB* (5’-TCGGTGTCGAGTACCGTCTCT-3’ and 5’-TGCCTTGCCGGTTGCTT-3’), *carRA* (5’-CAGAAGCTGTTCCCGAAGACA-3’ and 5’-TGCGATGCCCATTTCTTGA-3’), *carT* (5’-CGGCACCAACACCAGACA-3’ and 5’-TGGACTAGGAATGGCAAGGACTT-3’), *carX* (5’-GCCGCCCATGAGGATACA-3’ and 5’-TCAGCTTCAACACCGTCGAA-3’), *carO* (5’-TGGGCAACGCAGTGACAT-3’ and 5’-TGCGCAGACAGCCCAGTA-3’), *wcoA* (5’-TGAGATTGTCGGCCAGAATTG-3’ and 5’-GAGCCCGCTTCGACTTTG-3’), *cryD* (5’-CGGGACTACATGCGATTGTG-3’ and 5’- CTTGAAAAGACGTGAGCCAAACT-3’), and *vvdA* (5’-GCACCACCAGGGCATGA-3’ and 5’-GCGGTGTGAAGCGACCTT-3’). β-tubulin gene (5’-CCGGTGCTGGAAACAACTG-3’ and 5’-CGAGGACCTGGTCGACAAGT-3’) was used as an internal control to normalize the amount of cDNA in each reaction.

### Search of double Δ*cryD* Δ*wcoA* mutants

Different strategies were followed two obtain double mutants of the photoreceptor genes *cryD* and *wcoA*. Plasmid pDMWC was obtained from pALEX7 [27] by replacing the hygromycin resistance cassette by the *F*. *fujikuroi niaD* gene (GenBank X90699) as a selectable marker. A spontaneous *niaD* ^-^ mutant was obtained from the Δ*cryD* mutant SF237 by chlorate resistance as described [54]. Protoplasts of the *niaD* ^*-*^ Δ*cryD* strain were transformed with 30 μg of *Spe*I-linearized pDMWC and transformants were selected on DG medium, which contains nitrate as nitrogen source. Biolistic transformation was achieved with a Biolistic PDS-1000/He Particle Delivery System (Biorad, Hercules, CA, USA). 15 μg of plasmid pDMWC were shot on 10^6^ conidia on DG plates. For selection with an alternative resistance marker, the hygR cassette from pALEX7 [27] and pDcry [33] were replaced by the *nptII* cassette (neomycin phosphotransferase II) from plasmid pNTP2 [33] to yield plasmids pDwc2 and pGcry7, respectively. The occurrence of double recombination events was expected to generate Δ*wcoA* mutants with pDwc2 and Δ*cryD* mutants with pGcry7. To generate double Δ*cryD* Δ*wcoA* mutants, five transformations were performed incubating protoplasts of the Δ*cryD* mutant SF236 with pDwc2, and two transformations were performed with the opposite combination, incubating protoplasts of the Δ*wcoA* mutant SF226 with pGcry7.

Mutagenesis experiments of the *ΔcryD* mutants SF236 and SF237 were achieved either by UV-radiation or by exposure to N-methyl-N’nitro-N-nitrosoguanidine (NG) following standard protocols [53]. Plates with 10^3^ irradiated or NG-treated conidia were incubated in the dark for 3 days and screened for the formation of Δ*wcoA*-like purple-pigmented colonies in the dark.

### Constitutive *cryD* expression

*The cryD* coding region was expressed in the Δ*wcoA* mutant SF226 under control of the constitutive promoter of the glyceraldehyde-3-phospate dehydrogenase gene of *A*. *nidulans* (*gpdA*). For this purpose, plasmid pOEcry (9.6 kb), containing a tagged version of the *cryD* gene under control of the *gpdA* promoter and *trpC* terminator was generated. The coding region of the *cryD* gene was obtained by PCR on *F*. *fujikuroi* cDNA template with primers crycDNA-1F (5’-GGTACCTGGGAATAAGCTCCTCGTCTATC-3’) and crycDNA-1R (5’-GCGGCCGCATGGGGTCCAAGGTGAGGAGG-3’), modified to contain *Kpn*I and *Not*I restriction sites. These and other PCR reactions were performed with Expand High fidelity polymerase (Roche, Mannheim, Germany). The product was cloned into pGEM-T (Promega, Mannheim, Germany), the resulting plasmid was digested with *Kpn*I and *Not*I, and the corresponding fragment was cloned in pET51b+ (Novagen, Darmstadt, Germany), a plasmid used for protein targeting with Strep-tag in the N-term and a His_10_-tag in the C-term. The 2.2 kb sequence of the tagged *cryD* gene was amplified from the resulting plasmid with primers crytag-3F (5’-GCAGGATCCTAGGTTAATTAGT-3’) and crytag-3R (5’-GAAGGAGATATACTAGTGCAAG-3’), which include a *Bam*HI and a *Spe*I site, respectively. A reverse PCR was performed on pAN7–1 plasmid [55] to remove the *hph* coding region of the hygromycin resistance cassette with primers pAN7–1–3F (5’-GACCGCGGGTCCACTTAA-3’), which includes a *Bam*HI site, and pAN7–1–3R (5’-GGGAAATACTAGTTCTTGGATGG-3’), which includes a *Spe*I site. The 5.6 kb PCR fragment was digested with *Bam*HI and *Spe*I and ligated with the 2.2 kb *cryD Bam*HI/*Spe*I fragment, resulting in a 7.8 kb plasmid. pOEcry was obtained introducing in the *Eco*RI site of this plasmid a geneticin resistance cassette (1.8 kb) obtained by *Eco*RI digestion of the PCR product obtained from plasmid pNTP2 [33] with primers neo-2F (5’-CTCGTCTACTCCAAGAATTCC-3’) and neo-3R (5’-TCTAGAACTAGTGGATCCCC-3), which include *Eco*RI sites.

To obtain the *cryD*-overexpressing strain, 30 μg of pOEcry were used to transform SF226 protoplast according to Proctor et al. [56], and the transformants were selected on a medium supplemented with geneticin (G418 disulfate salt, Sigma) as described [33]. The presence of the geneticin resistance cassette in the transformants was confirmed by PCR with primers neo-3F (5’- GAACAAGATGGATTGCACGC-3’) and neo-4R (5’-CGCTCAGAAGAACTCGTCAA-3’), which amplify the 0.8 kb *nptII* coding region. The presence of the P*gpdA*-*cryD* sequence was verified by PCR with primers gpd-2F (5’-ggctcaaatcaataagaagaacgc-3’) and cry-4R (5’-CATTCAGCTCCGTAGCGC-3’), which yield a 3.9 kb PCR fragment.

### Carotenoid analyses

Mycelial samples were separated from the agar cultures with a sterile blade, frozen at -20°C and freeze-dried for 24 h in a VirTis SP Scientific sentry 2.0 equipment (SP Industries, Warminster, PA, USA). The dry samples were weighed and disrupted in a FAST-PREP 24 device (MP biomedicals, Irvine, CA) or in a Precellys 24 homogenizer (Bertin Technologies, Montigny le Bretonneux, France) with 1 ml of acetone as described [57]. Extractions were repeated up to total bleaching of the samples (3–6 extractions depending on carotenoid content) and the extracted solvent was dried in a Concentrator Plus equipment (Eppendorf, Hamburg, Germany). Dry samples were resuspended either in 1 ml or 0.1 ml hexane, depending on carotenoid concentrations, and subjected to spectrophotometrical determinations in the range of 350–650 nm (Shimadzu UV spectrophotometer 1800). Maximal absorbance at 482 nm was used to estimate the total amount of carotenoids, based on an average maximal E (1 mg l^-1^, 1 cm) of 200, and normalized according to the dried weight and dilution of the sample. When required (i.e., in analyses of Δ*cryD* or Δ*wcoA* mutants), bikaverins were removed from the carotenoid fraction as described [27].

### Statistical analyses

To check if differences between different data values were significant, a statistical analysis was performed with the GraphPad Prism program (GraphPad Software, Inc. La Jolla, CA, USA). An ANOVA test (ANalysis Of VAriance) to compare several groups at the same time was used. The threshold of significance was set to a p value of 0.05 and three levels of significance were distinguished: p< 0.05, 0.05>p>0.001 and p<0.001. In the case of statistical significance (at least one group has a different average value), a Bonferroni post-correction test was applied to determine to which particular times and strains these differences were due in comparison to the wild type. The results of the statistical analysis are displayed in S1 Table.

## Supporting Information

S1 Fig Generation and analysis of *cryD* overexpression in a Δ*wcoA* mutant. A. Left: Schematic representation of the relevant segment in plasmid pOECry containing the *cryD* gene under control of the *A*. *nidulans gpdA* promoter. The geneticin resistance marker *nptII* is indicated in yellow. Colored arrowheads indicate the primers used in the analysis of the transformant. Right: Agarose gel electrophoresis of PCR amplification products obtained from DNA samples of the Δ*wcoA* mutant SF226 and the SF226-derived *cryD* overexpressing strain (oe) with the primers sets indicated under the picture. M: Markers. Relevant sizes of markers and PCR products are shown in kb. B. Real-time RT-PCR analyses of the genes *cryD* (left panel), *carRA* (central panel) and *carB* (right panel) in RNA samples of the wild type, the Δ*wcoA* mutant SF226 and the SF226-derived *cryD* overexpressing strain (oe). For each strain, the left bar (dark color) corresponds to 3-day incubation in DGasn medium in the dark and the right bar (pale color) stands for one hour of illumination. Relative expression for each gene was referred to the value in the wild type grown in the dark. Data are the means and standard deviations of six determinations from two biological replicates. C. Kinetics of carotenoid accumulation after illumination of the Δ*wcoA* mutant SF226 and the SF226-derived *cryD* overexpressing strain (oe). The strains were incubated for three days in the dark on DGasn agar and exposed to white light for the time indicated in abscissae. Each point is the mean and standard deviation of four determinations from two biological replicates.
(PDF)

S2 Fig Aspect of the colonies of the wild type and the Δ*wcoA* (SF226 and SF229), Δ*cryD* (SF236 and SF237), and Δ*vvdA* (SF256 and SF258) mutants used for the carotenoid analyses described in Fig. 3. The strains were incubated for three days in the dark in DGasn medium and exposed to 0.07 W m^-2^ (1%), 0.7 W m^-2^ (10%) or 7 W m^-2^ (100%) of white light for 6 hours (above) and 48 h (below). Relative positions of the strains are schematized on the left.
(PDF)

S1 Table Statistical analyses. Strains and conditions in Figs. 2, 4, 5 and 7 for which the differences with the equivalent data in the wild-type strain were statistically significant according to the ANOVA test (see Materials and Methods).
(PDF)
