# Supplementary figures and images for: The Flavoproteins CryD and VvdA Cooperate with the White Collar Protein WcoA in the Control of Photocarotenogenesis in Fusarium fujikuroi
Source: PLoS One. 2015 Mar 16;10(3):e0119785. doi: 10.1371/journal.pone.0119785 (PMC4361483; doi:10.1371/journal.pone.0119785)

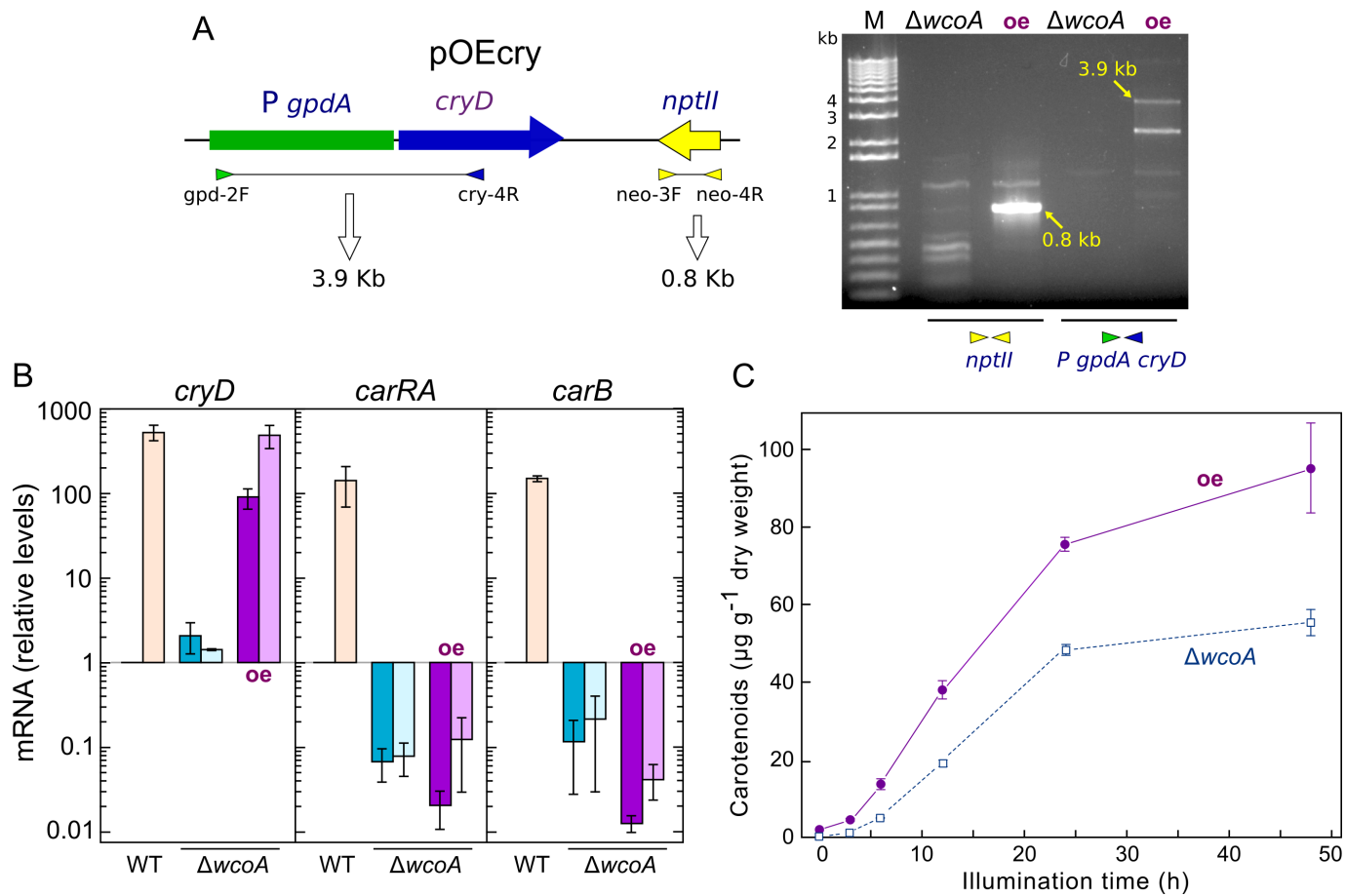

Supplement: S1 Fig — A. Left: Schematic representation of the relevant segment in plasmid pOECry containing the cryD gene under control of the A. nidulans gpdA promoter. The geneticin resistance marker nptII is indicated in yellow. Colored arrowheads indicate the primers used in the analysis of the transformant. Right: Agarose gel electrophoresis of PCR amplification products obtained from DNA samples of the ΔwcoA mutant SF226 and the SF226-derived cryD overexpressing strain (oe) with the primers sets indicated under the picture. M: Markers. Relevant sizes of markers and PCR products are shown in kb. B. Real-time RT-PCR analyses of the genes cryD (left panel), carRA (central panel) and carB (right panel) in RNA samples of the wild type, the ΔwcoA mutant SF226 and the SF226-derived cryD overexpressing strain (oe). For each strain, the left bar (dark color) corresponds to 3-day incubation in DGasn medium in the dark and the right bar (pale color) stands for one hour of illumination. Relative expression for each gene was referred to the value in the wild type grown in the dark. Data are the means and standard deviations of six determinations from two biological replicates. C. Kinetics of carotenoid accumulation after illumination of the ΔwcoA mutant SF226 and the SF226-derived cryD overexpressing strain (oe). The strains were incubated for three days in the dark on DGasn agar and exposed to white light for the time indicated in abscissae. Each point is the mean and standard deviation of four determinations from two biological replicates. (PDF) [file pone.0119785.s001.pdf]
