# Supplementary material for: The Flavoproteins CryD and VvdA Cooperate with the White Collar Protein WcoA in the Control of Photocarotenogenesis in Fusarium fujikuroi
Source: PLoS One. 2015 Mar 16;10(3):e0119785. doi: 10.1371/journal.pone.0119785 (PMC4361483; doi:10.1371/journal.pone.0119785)
Supplement: S2 Fig — The strains were incubated for three days in the dark in DGasn medium and exposed to 0.07 W m-2 (1%), 0.7 W m-2 (10%) or 7 W m-2 (100%) of white light for 6 hours (above) and 48 h (below). Relative positions of the strains are schematized on the left. (PDF) [file pone.0119785.s002.pdf]

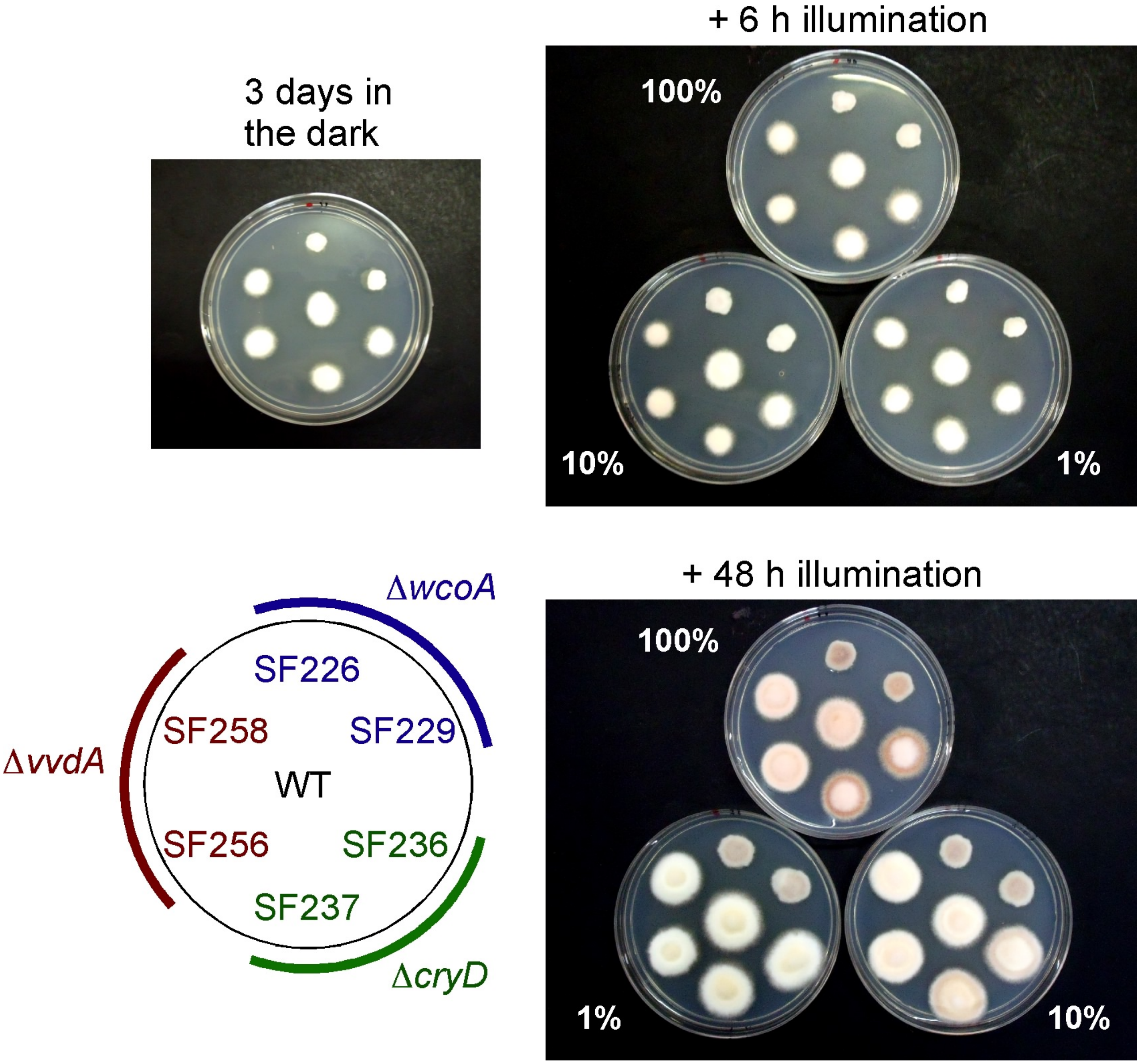

S2 Fig. Aspect of the colonies of the wild type and the Δ*wcoA* (SF226 and SF229), Δ*cryD* (SF236 and SF237), and Δ*vvdA* (SF256 and SF258) mutants used for the carotenoid analyses described in Fig. 3.
