# Supplementary material for: The Flavoproteins CryD and VvdA Cooperate with the White Collar Protein WcoA in the Control of Photocarotenogenesis in Fusarium fujikuroi
Source: PLoS One. 2015 Mar 16;10(3):e0119785. doi: 10.1371/journal.pone.0119785 (PMC4361483; doi:10.1371/journal.pone.0119785)
Supplement: S1 Table — Strains and conditions in Figs. 2, 4, 5 and 7 for which the differences with the equivalent data in the wild-type strain were statistically significant according to the ANOVA test (see Materials and Methods). (PDF) [file pone.0119785.s003.pdf]

S1 Table. Statistical analyses.

Fig. 2B

Significance level	*carB*	*carRA*
$p < 0.05$	SF226 L<br>SF236 L<br>SF237 L	SF226 D<br>SF229 D
$0.05 > p > 0.001$	SF226 D<br>SF229 D	

Fig. 4

Significance level	Dark	6h 100%	6h 10%	6h 1%	48h 100%	48h 10%	48h 1%
$p < 0.05$	SF258	SF236					SF237
$0.05 > p > 0.001$	SF226<br>SF229		SF236				SF236
$p < 0.001$	SF256	SF226<br>SF229<br>SF256<br>SF258	SF226<br>SF229<br>SF256<br>SF258	SF226<br>SF229<br>SF256<br>SF258	SF226<br>SF229<br>SF236<br>SF237<br>SF256<br>SF258	SF226<br>SF229<br>SF236<br>SF237	SF226<br>SF229

Fig. 5

Significance level	*cryD*	*wcoA*
$p < 0.05$	SF258 0’	SF257 240’
$0.05 > p > 0.001$	SF258 120’	SF258 0’<br>SF258 240’
$p < 0.001$	SF258 60’<br>SF258 30’<br>SF258 60’	

Fig. 7

Significance level	*carB*	*carRA*	*carT*	*carO*	*carX*
$p < 0.05$	SF237 30’	SF237 120’			SF236 30’
$p < 0.001$	SF237 60’<br>SF237 60’	SF236 0’<br>SF237 0’<br>SF236 30’<br>SF237 30’<br>SF236 60’<br>SF237 30’	SF237 0’<br>SF237 30’	SF237 60’	SF237 30’<br>SF236 60’<br>SF237 30’
